# Constrictive Pericarditis Associated with Atypical Antipsychotics

**DOI:** 10.1155/2012/805939

**Published:** 2012-07-04

**Authors:** Kuan-chin Jean Chen, Aashish Goela, Patrick Teefy, L. Ray Guo

**Affiliations:** ^1^Division of Cardiac Surgery, Department of Surgery, University of Western Ontario and Lawson Health Research Institute, London Health Sciences Centre, London, ON, Canada N6A 5A5; ^2^Department of Family Medicine, University of Western Ontario, London, ON, Canada N6A 5C1; ^3^Department of Radiology, University of Western Ontario and London Health Sciences Centre, London, ON, Canada N6A 5A5; ^4^Division of Cardiology, Department of Internal Medicine, University of Western Ontario and London Health Sciences Centre, London, ON, Canada N6A 5A5; ^5^Department of Surgery and University Hospital, London Health Sciences Centre, 339 Windermere Road, London, ON, Canada N6A 5A5

## Abstract

We report the successful surgical intervention in a case of constrictive pericarditis after long-term use of atypical antipsychotics. Pericarditis developed in our patient with a longstanding history of schizophrenia treated with atypical antipsychotics. Pericardiectomy was undertaken, and the patient's presenting symptom of shortness of breath resolved subsequently with an uneventful postoperative course.

## 1. Case Report 

**Figure 1:**
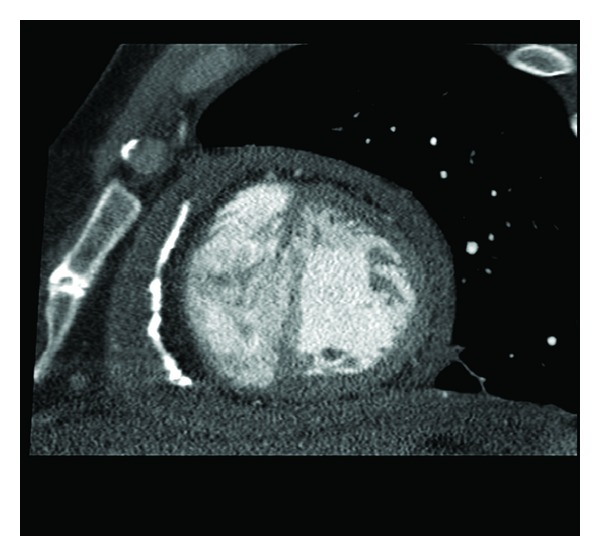
CT image indicating pericardial thickening.

**Figure 2:**
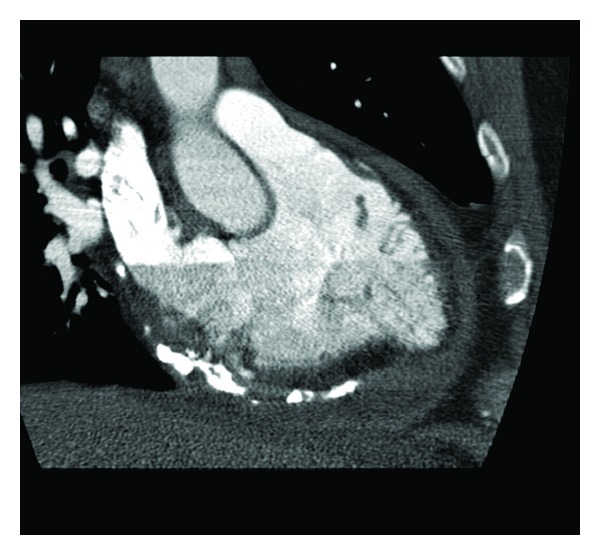
Pericardial calcification.

A 42-year-old man presented with history of severe dyspnea and fatigue along with increasing abdominal girth and pedal edema. The patient had an approximately ten-year history of schizophrenia. Following his hospitalization seven years ago for psychosis and auditory hallucination, he went on trials of medication; his schizophrenia was ultimately stabilized with long-term use of quetiapine at 900 mg once daily. After presenting with severe shortness of breath, he was admitted under cardiology. Transthoracic echocardiogram revealed moderate-sized pericardial effusion with signs of pericardial constriction. He underwent pericardiocentesis with some improvement in symptomatic shortness of breath. However, there was gradual re-accumulation of pericardial effusion with continuation of quetiapine. Subsequent chest X-ray and thoracic CT scan revealed calcification of the pericardium (Figures 1 and 2). Cardiac catheterization revealed normal coronary arteries and hemodynamics consistent with pericardial constriction. The patient then underwent pericardiectomy. The pericardium was found to be thickened and firmly adherent to the epicardium of the right and left ventricles. The pericardium was also adhering to the ascending aorta. There were spicules of calcification over the anterior wall of the right ventricle, which invaded through the epicardium into the myocardium. The pericardium overlying the inferior vena cava and right pulmonary veins was found to be ossified. Pericardiectomy was undertaken with the pericardium submitted for histopathologic and microbiologic examination, which revealed fibrously thickened pericardium lined by fibrinous exudate and focal calcification without granulomatous acute inflammation or malignancy. Following pericardiectomy, the patient's dyspnea resolved and had an uncomplicated course during his stay in the hospital. Quetiapine was subsequently tapered, discontinued and substituted to aripiprazole with no recurrence in symptoms and no re-accumulation of pericardial effusion. 

## 2. Comments 

The typical clinical presentation of acute pericarditis includes pleuritic-type chest pain. Pericarditis may result from radiation therapy, myocardial infarction, malignancy, systemic diseases such as systemic lupus erythematosus and hypersensitivity reaction, or infection such as tuberculosis and Coxsackie B. Coxsackie B is a common cause of pericarditis but is usually not associated with calcification and symptomatic constriction. On the other hand, tuberculosis is a common cause of pericardial effusion followed by constriction and calcification. While sometimes the etiology of pericarditis is evident, more often the diagnostic yield of pericarditis is low. Permanyer-Miralda et al. conducted a prospective series of 231 consecutive patients with primary acute pericardial disease and found that only in 14% of patients a specific etiologic diagnosis was obtained. Moreover, the diagnostic yield for pericardiocentesis was only at 6% [1]. 

There are numerous mechanisms in which a drug can induce pericardial disease. Medications such as procainamide and isoniazid induce pericarditis via lupus erythematosus reaction. Penicillin and tryptophan can induce a hypersensitivity reaction leading to pericardial disease. Antitetanus or other blood products can cause serum sickness, and medications such as cyclosporine, 5-FU, amiodarone, thiazide, and sulfa can cause an idiosyncratic reaction or hypersensitivity, further causing pericarditis [2]. 

We are aware of reported cases of pericarditis induced by clozapine [3, 4]. Murko et al. described a case of pericardial effusion and pericarditis in a man with chronic paranoid schizophrenia that had been stabilized with seven-year use of clozapine [3]. Daly et al.'s report described re-accumulation of pericardial fluid with the continuation of clozapine. However, other atypical antipsychotic agents such as quetiapine-mediated constrictive pericarditis have not been reported in the published literature. 

Atypical antipsychotic medications were introduced in the late 1980s with the distinctive characteristic in their low risk of extrapyramidal symptoms (EPSs) and tardive dyskinesia [5]. In our case, quetiapine was the chosen agent for the individual patient response and its low incidence of EPS at higher doses, as prescribed in our patient [5]. However, it is known to be associated with less than 1% risk of myocarditis. In our patient, all possible causes of pericarditis were ruled out with the exception of viral infection. We did not seek the evidence of the viral infection as the patient presented with no infectious symptoms, in addition to the time delay and inaccuracy of viral titers and limited implications of clinical management. Furthermore, the re-accumulation of pericardial effusion with the continuation of quetiapine further suggests that the patient's pericarditis was quetiapine induced. 

In conclusion, as accumulation of pericardial effusion requires prompt treatment and a high clinical suspicion, it is important for the clinician to be cognizant of quetiapine-induced pericarditis and to discontinue the causative agent if a patient develops clinically significant pericarditis. In the case of symptomatic pericardial constriction, pericardiectomy may be needed and is able to provide satisfactory outcome as evidenced in our case.
